# A preliminary study of clinical assessment of left unilateral spatial neglect using a head mounted display system (HMD) in rehabilitation engineering technology

**DOI:** 10.1186/1743-0003-2-31

**Published:** 2005-10-05

**Authors:** Toshiaki Tanaka, Shunichi Sugihara, Hiroyuki Nara, Shuichi Ino, Tohru Ifukube

**Affiliations:** 1 Department of Physical Therapy, School of Health Sciences Sapporo Medical University, Sapporo, Hokkaido, Japan; 2 Sapporo Shuyukai Hospital, Sapporo, Hokkaido, Japan; 3 AdIn Research, Inc., Sapporo, Hokkaido, Japan; 4 Research Center for Advanced Science and Technology, The University of Tokyo. Tokyo, Japan

**Keywords:** Unilateral spatial neglect, head mounted display system, virtual reality, clinical assessment

## Abstract

**Purpose:**

Unilateral spatial neglect (USN) is a common syndrome in which a patient fails to report or respond to stimulation from the side of space opposite a brain lesion, where these symptoms are not due to primary sensory or motor deficits. The purpose of this study was to analyze an evaluation process system of USN in various visual fields using HMD in order to understand more accurately any faults of USN operating in the object-centred co-ordinates.

**Method:**

Eight stroke patients participated in this study and they had Left USN in clinical test, and right hemisphere damage was checked by CT scan. Assessments of USN were performed the BIT common clinical test (the line and the stars cancellation tests) and special tests the zoom-in condition (ZI) condition and the zoom-out condition (ZO) condition. The subjects were first evaluated by the common clinical test without HMD and then two spatial tests with HMD. Moreover, we used a video-recording for all tests to analyze each subject's movements.

**Results:**

For the line cancellation test under the common condition, the mean percentage of the correct answers at the left side in the test paper was 94.4%. In the ZI condition, the left side was 61.8.% and the right side was 92.4.%. In the ZO condition, the left side was 79.9% and the right side was 91.7.%. There were significant differences among the three conditions. The results of the stars cancellation test also showed the same tendency as the line bisection test.

**Conclusion:**

The results showed that the assessment of USN using a technique of HMD system may indicate the disability of USN more than the common clinical tests. Moreover, it might be hypothesized that the three dimensional for USN test may be more related to various damage and occurrence of USN than only the two dimensional test.

## Introduction

Unilateral spatial neglect (USN) is a common syndrome in which a patient fails to report or respond to stimulation from the side of space opposite a brain lesion, where these symptoms are not due to primary sensory or motor deficits [1]. Patients with severe neglect often collide with objects, ignore food on one side of the plate, and in general tend to rely on just one side of the body [2]. Patients with USN of the left hemispace require longer hospital stays and have more difficulty resuming activities of daily living [3]. Katz et al. [4] reported that impairment and disability levels of RBD patient with and without USN were clearly different. Neglect is associated with lower performance on measures of impairment, as well as on measures of disability in ADL. Recently, several studies have singled out USN as one of the major disruptive factors impeding functional recovery and rehabilitation success [5].

Progress in the treatment of USN has been hampered by an inadequate understanding and examination of the underlying involved mechanisms [6]. One problem has been the underrepresentation of left hemisphere-damaged patients in many studies, despite several reports which indicated no significant differences in the frequency of neglect [7]. The situation is further complicated by the existence of competing theoretical models [8,9], different lesion locations, and considerable variation in the reported incidence among right-brain-damaged patients [10]. Little attention has been paid to systematic behavioral assessment of patients with USN. As a result, there has been a largely unquestioned assumption that the diverse assessment procedures all provide an accurate measurement of the same underlying deficit.

From a rehabilitation perspective, the traditional assessment of USN centers on a variety of simple perceptual motor tasks. Investigations have used line crossing [11], cancellation task [12] and more recently, an indented reading test [13]. However, there is no single standardized battery of tests currently available for the assessment of USN. Also, performance rating of these tasks cannot be related to the specific difficulties encountered in everyday life. Rehabilitation prospects of brain-damaged patients are rendered more specific and realistic by a consideration of their behavioral strengths and deficits within a functional framework [14]. The development of an objective behavioral test of everyday skills relevant to neglect would provide therapists and clinicians with a more precise description of a patient's capabilities, which would encourage a more robust grounding for rehabilitation.

An analysis of USN can be explained with a space coordinate system theory. The boundaries of the neglected space are not constant in as much as the neglect patients'performance is influenced by the relevant system of spatial coordinates; egocentric or allocentric co-ordinates. Egocentric co-ordinates specify locations relative to the viewer [15], whereas allocentric co-ordinates code their position independent of viewpoint [16]. Clinical evidence from visuospatial neglect suggests that some patients neglect one side of each individual object in a scene, rather than just one side of the scene as a whole. For example, in copying a lateral array of objects, right-hemisphere patients may reproduce only the right side of the objects, but produce these for each of the objects in the scene including those on the extreme left [17]. This is suggestive evidence for neglect operating in the object-centred allocentric co-ordinates. Driver and Halligan suggested that USN can be object-centered in the sense of operating relative to the principal axes [18]. However, copying evidence is not conclusive.

Several sensory manipulations may be temporarily effective for improving unilateral spatial neglect. Karnath indicated the effectiveness of neck vibration [19]. Pizzamiglio et al. also adopted an effective means of optokinetic stimulation [20]. Rossetti et al. investigated the effect of prism adaptation on neglect symptoms, including the pathological shift of the subjective midline to the right [21]. They reported that all patients exposed to the optical shift of the visual field to the right were improved in their manual body-midline demonstration and on their classical neuropsychological tests. However, these manipulations have not yet succeeded in bringing about a consistent improvement of neglect.

Virtual reality (VR) refers to computer-generated, usually visual, representation of real-world objects in which a user can navigate or manipulate the environment [22]. The most well-known approach is " immersive, " where the real world is opaque to the user and he or she is provided the sensation of interacting directly with the computer-generated objects. In other approaches, VR shares certain attributes similar to a three-dimensional computer-aided design (CAD). In immersive VR, a head-mounted display (HMD) is worn and its position in space is tracked. As the user moves his or her head, aspects of the computer-generated object appropriate to the HMD position are displayed. Virtual reality (VR) has many advantages over other ADL rehabilitation techniques and offers the potential to develop a human performance testing and training environment [23] and also a VR system for training individuals with unilateral spatial neglect to cross streets in a safe and vigilant manner. [24]. VR can give human versatile sensory information artificially and easily for the visual, vestibular, and the somatic sensations. Recently, VR has been investigated in a few studies using devices for compensation of visual sensory. For example, there is one approach where HMD gives a patient with Parkinson' disease an emphasized visual input in order to improve a frozen gait of the patient [25]. HMD has a function which can focus on a certain object or to limit the surrounding environmental conditions, and to offer versatile visual information. Therefore, HMD can produce the object-centred co-ordinates for a USN patient.

The purpose of this study was to analyze an evaluation process system of USN in various visual fields using HMD in order to understand more accurately any faults of USN operating in the object-centred co-ordinates. Moreover, we constructed a new device that uses rehabilitation engineering technology for assessing and training of USN.

The following hypothesis was verified that a special evaluation process system with HMD for USN can be more accurate and detailed than the common clinical test for USN. It may be assumed that the significant difference between the common evaluation of USN and the special test in the object-centred co-ordinates was produced by the result of using HMD.

However, there were a few limitations of this study. There was the possibility of low validity of the results because of the small number of subjects. There was also a limitation about discussion of concerning the mechanism of USN because of the damaged part of the brain and the versatility of coping mechanisms.

## Methods

### 1. Subjects

Eight patients who had suffered a stroke (mean age 67.1 years old) participated in this study after gaining their informed consent. The patients were tested for the presence of any neglect for activities of daily living (ADL) by two therapists. Two medical doctors checked the right hemisphere damage of all subjects by CT (computed tomography) or MRI (magnetic resonance imaging). Individuals with weak visual acuity, dementia, hemianopsia, apraxia or those being left-handed were excluded. The subjects could sit on an ordinary chair by themselves. The period from the appearance of disease to study assessment was 4–27 weeks (Table 1).

**Table 1:** Characteristics of patients

Patient No.	Age (years)	Dignosis	Lesion*	Time of rehabilitation onset (weeks)	FIM-M
1	75	I	FTP	6	30
2	65	I	BgFPT	1	38
3	64	H	Th	1	61
4	63	H	Bg	1	35
5	56	I	PT	1	85
6	70	I	Bg	1	33
7	79	I	FPT	1	86
8	68	I	BgFPT	2	72

Abbreviations: I: infarction, H; hemorrhage, F: frontal lobe, P: parietal lobe; T; temporal lobe, Bg; basal ganglia, Th; thalamus. FIM; Functional Independence measure Motor.

*all lesions were right sided.

### 2. Functional assessment

The Functional Independence Measure (FIM) was executed as an ADL evaluation [26,27]. The FIM motor sub scores (FIM-M) was used for measure of disability as the best predictors of rehabilitation length of stay for stroke. Moreover, two therapists evaluated the patients who exhibited specific neglect behaviors in ADL using a special checklist (Table 2). The checklist used a modified version of Halligan's checklist [28]. The therapists were requested to score the checklist in terms of those behaviors they considered to be related to as visual neglect, as opposed to poor performance that might be expected to follow concomitant disorders such as problems of motor coordination or initiation.

**Table 2:** Checklist of Everyday Neglect Behaviors

1. Dose the patient show difficulties when: talking or communicating with others
2. Dose the patient neglect the left/right side of personal space?
3. Dose the patient show difficulties in eating?
4. Dose the patient show difficulties in grooming (self-care skills, washing, bathing, etc)
5. Does the patient show difficulties in dressing?
6. Does the patient show difficulties in body movement transferring (from a bed to W/C,etc)?
7. Does the patient show difficulties in locomotion 1 (the patient collides against objects and wall on the affected side. The patient can not negotiate a W/C between doors, kerbs, etc.)?
8. Does the patient show difficulties in locomotion 2 (the patient turns toward the direction of the affected side.)
9. Does the patient show difficulties during PT exercise?
10. Does the patient show difficulties during OT excercise?

### 3-1. Evaluation for USN

#### 3-1-1. Common clinical test (Figure 1)

**Figure 1:**
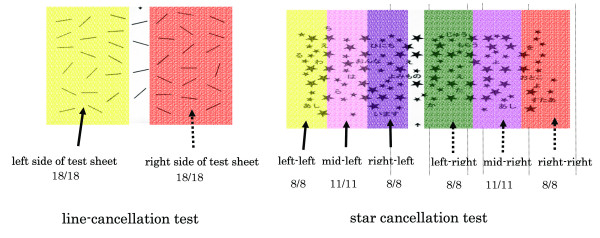
Analysis method for line and star cancellation test.

To asses neglect, the widely used line and star cancellation tests as included in the Behavioral Inattention Test (BIT) were given to the subjects [29]. We used the BIT Japanese version which was modified by Ishiai et al [30].

For the line cancellation test (score range from 0 to 36 points), the subjects were presented with a single sheet of paper on which 6 lines in varying orientations were drawn, 18 on each side. They were instructed to make a mark through all of the lines. Left- sided neglect was indicated by a failure to mark more lines on the left side than on the right. Degree of neglect was assessed by the proportion of lines omitted relative to the total number of lines. The line cancellation test sheet was divided into right and left portions and a right and then a left correct answer rates were analyzed. 34 points were set as a cutoff value.

For the star cancellation test (score range from 0 to 54 points), the A4 stimulus sheet contained 56 targets (small stars) pseudo-randomly interspersed with distracter items. The targets actually fell into six columns, with two additional targets which were located centrally. The experimenter clearly indicated the full extent of the sheet and crossed out the two central targets as an example to the subject. The subject was then asked to cancel the remaining small stars. The number of targets omitted in each lateral half of the sheet was counted. The star cancellation test sheet was divided into six areas (left-left, middle-left, right-left areas and right-right, middle-right, left-right areas) and was analyzed using the correct rate for six areas. 51 points were set as a cutoff value.

### 3-2. Special test with HMD (Figure 2)

**Figure 2:**
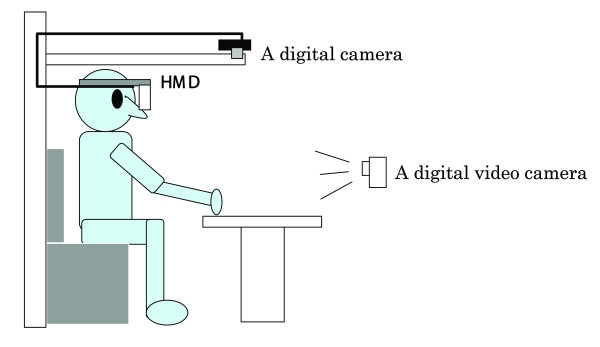
Experimental setup for the HMD (head mounted display) system.

#### (a) Experimental apparatus

The main experimental apparatus includes a digital camera, HMD (GT270, Canon Inc.), and a digital video camera. HMD is a glass type display method (270,000 pixel, effective pixel number is 99.99%, weight is 150 g) that consists of two TFT liquid crystal panels. The digital camera takes a picture of a test sheet on the desk, and HMD presents the subject from the digital camera. Moreover, the subject's head movement was recorded by a digital video camera as a qualitative motion analysis.

#### (b) Assessments of USN with HMD (Figure 3)

**Figure 3:**
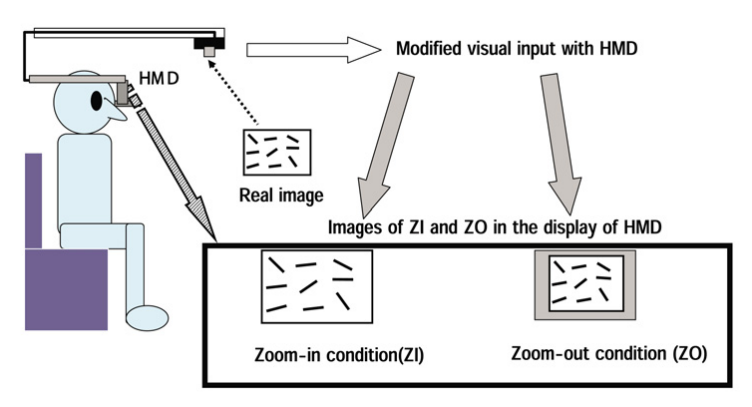
Two special tests of USN with HMD.

We attempted to find the degree that USN alters when the co-ordinate of the subject's visual field was carried out as object-centered by HMD. Therefore, we used two different lens of the digital camera in order to change visual field and then HMD displayed the test paper to the subject as the two special tests as follows;

1) Special test 1: the zoom-in (ZI) condition which can display only the test paper using combined HMD and a DV camera.

2) Special test 2: the zoom-out (ZO) condition which can display 0.7 times special condition1 by changing the lens.

### 3-3. Procedure

The subjects sat on a wheelchair if needed or a straight back chair sitting in an up-right position as a starting point. The test paper on a desk was placed at a midline of each subject's body. All tasks were done without any restriction as to time.

The subjects were first evaluated by a normal test without HMD as the common clinical test and then two spatial tests with HMD. The line cancellation test was scored using the correct rate and then the score divided into two areas; right and left. The star cancellation test was scored using the correct rate for six areas (left-left, middle-left, right-left areas and right-right, middle-right, left-right areas) in which the test paper was divided (Figure 1). All subjects performed in random order the common clinical test and two special tests (ZI, ZO). The examiner confirmed the HMD monitor as the display from the image of the digital camera. Moreover, the movements of head, trunk, and upper/lower extremities were were qualitatively analyzed during these tests for finding an abnormal movement.

### 4. Data analysis

All statistics were performed using SPSS statistical software (7.5.2 J). An ANOVA or Student's t test was used as a comparison between the common clinical test and the two special tests with HMD. Moreover, a Student's t test or an ANOVA was used for a comparison within the line cancellation test and the star cancellation test, respectively. Multivariate ANOVA tests were performed in each group and Shėffe post hoc tests were performed if significant differences were found at the 5 % significance level.

The qualitative analysis of head, trunk, and upper/lower extremity movement during all tests was performed by the digital video camera in a sagittal or a frontal plane.

## Results

In this study, the average of FIM-M of all subjects was 53.0 ± 21.6 points (Table 1). The subject needs maximal or moderate assistance for some performance of ADL.

As the common clinical test for USN, in the first evaluation of the frequency of presence of neglect for ADL (Table 3), 75 percent of all subjects admitted a USN symptom in activities of dressing. For example, a patient with USN cannot easily put on their clothes on the left side. Moreover, 62.5 percent of the subjects admitted a USN symptom in activities of transferring, and locomotion (Table 3). According to the motion analysis of head motion in the common clinical test, the subjects began searching from the right side in both the line and the star cancellation tests. In a normal performance, the head naturally rotated from the right to the left to follow a movement during the line cancellation test. However, the head movement to their left was insufficient for searching from the right side in the both tests. For the line cancellation test under the common condition, the mean percentage of the correct answers at the left side in the test paper was 94.4%. The right side was 100 %. Nobody fell below the cutoff value (Table 4) [30]. For the star cancellation test under the common clinical test (Table 5), the mean percentage of the correct answers at the left- left area was 91.1 %. The middle-left area was 89.2 % and the right-left side was 84.4 %. The mean percentage of the correct answers at the right-right was 92.9 %, middle-right was 96.4 %, and left-right area was 81.8 %. Three subjects fell below the cutoff value as an abnormal [30].

**Table 3:** Ratio of USN symptoms in ADL

	n = 8	Ratio of USN (%)
talking or communicating with others	4	50.0
neglecting the left side of bed space	2	25.0
eating	1	12.5
grooming (self-care skills, washing, bathing,etc)	2	25.0
dressing	6	75.0
transferring (from a bed to W/C.etc)	5	62.5
lecomotion 1 negotiatin a W/C between doors, kerbs, etc.	5	62.5
lecomotion 2 the patient turns toward the direction of the affected side.	5	62.5
during PT exercise	6	75.0
during OT excercise	7	87.5

**Table 4:** Mean percentage of correct answers of the line cancellation test in the common method.

	Mean percentage of correct answers (%)
left side of test sheet	95.1 ± 13.8
right side of test sheet	100 ± 0

**Table 5:** Mean percentage of correct answers of the star cancellation test in the common method.

	Percentage of correct answers (%)
Correct answers of left-left	91.1 ± 13.7
Correct answers of right-left	81.8 ± 31.1
Correct answers of mid-left	89.3 ± 8.6
Correct answers of mid-right	96.4 ± 5.9
Correct answers of left-right	84.4 ± 30.1
Correct answers of right-right	92.9 ± 14.0

For the special test with HMD, in the motion analysis of head motion, the subjects began searching from the right side in both the line and the star cancellation tests. However, seven subjects kept rotating only on the right side. They did not rotate to the left side. For the line cancellation test under the ZI condition in the special test with HMD (Table 6), the mean percentage of the correct answers at the left side in the test paper was 61.8 %. The right side was 92.4 %. For the ZO condition, the mean percentage of the correct answers at the left side in the test paper was 79.9 %. The right side was 91.7 %. In both ZI and ZO conditions, the left score was significantly greater than the right score (p < 0.05). There was a significant difference between the common clinical test and ZI conditions of the special test with HMD for the left side score (p < 0.05). For the star cancellation test under the ZI condition in the special test with HMD (Table 7.), the mean percentage of the correct answers at the left- left area was 60.7 %. The middle-left area was 69.6 % and the right-left side was 77.9 %. The mean percentage of the correct answers at the right-right was 87.5 %, middle-right was 92.9 %, and left-right area was 87.0 %. For the ZO condition, the mean percentage of the correct answers at the left- left area was 69.7 %. The middle-left area was 70.8 % and the right-left side was 77.9 %. The mean percentage of the correct answers at the right-right was 97.9 %, middle-right was 87.5 %, and a left-right area was 92.4 %.

**Table 6:** Mean percentage of correct answers of the cancellation test in three conditions

	correct answers for left side (%)	correct answers for right side (%)
Common	95.1 ± 13.8^ab^	100 ± 0
ZI	61.8 ± 34.3^a^	92.3 ± 11.1
ZO	79.8 ± 37.6^a^	91.7 ± 14.5

^a ^significant difference between right and left (p < 0.05)

^b ^significant difference between common and ZI (p < 0.05)

**Table 7:** Mean percentage of correct answers of the star cancellation test in three conditions

	Mean percentage of correct answers(%)		
	Common	ZI	ZO
	
Correct answers of left-left	91.1 ± 13.7	60.7 ± 47.0	66.7 ± 51.6
Correct answers of right-left	81.8 ± 31.1	87.0 ± 10.2	69.7 ± 38.4
Correct answers of mid-left	89.3 ± 8.6	69.6 ± 37.4	70.8 ± 42.3
Correct answers of mid-right	96.4 ± 5.9	92.9 ± 6.4	87.5 ± 13.7
Correct answers of left-right	84.4 ± 30.1	77.9 ± 37.0	69.9 ± 38.4
Correct answers of right-right	92.9 ± 14.0	87.5 ± 14.3	97.9 ± 4.9

## Discussion

All subjects reported that the HMD presented a brighter, clearer image almost at real time and there was no discomfort in wearing the HMD. In this study, HMD can be shown as if the subject was looking at a 52 inch display screen 2 m away. Moreover, a change in the range of indirect vision field became possible by operating the input method using the HMD with a computer.

A digital camera was used for projecting the test sheet on the liquid crystal screen of the HMD. This camera was fixed, so that the test sheet reflected on the liquid crystal screen of HMD did not move, even if the head did during a test. This implies that the special test with HMD produced a better suited condition of the object-centred allocentric co-ordinates than the common condition test did. In this study, ZI condition was the same as that of the object-centred allocentric co-ordinates.

For motion analysis during the special test with HMD, the results showed that the subjects had the tendency to mainly focus on the right side of the test sheet under the conditions of ZI and ZO as compared to the common clinical test for USN. In a viewing the video recording as a qualitative motion analysis, when subject performed special test with HMD, there was a tendency that the subject tried to concentrate more on the right side of the test sheet. It may be that the subject's neglect was enhanced by HMD. Since the special test with HMD produced the object-centered allocentric coordinate, the subject focused more on the test sheet itself than the common clinical test. This means that if the subject pays too much attention to an object, it may be risk factor that he/she ignores the left side. Moreover, Ishiai et al. examined USN patient's eye movement using an eye camera [31]. The eye movement of a healthy person and the patients with homonymous hemianopia who have no USN symptoms could maintain a central focus. However, the patients with homonymous hemianopia who also have USN symptoms veered to the right side and their eyes did not move to the left side. HMD might be able to better clarify the left neglected area because the patients can concentrate on the object (test sheet) by limiting the viewing area as compared with the common clinical test.

The correct answer rate of the left space under ZI and ZO conditions was significantly lower than those in the common clinical test. Moreover, the correct answer rate which rose under the ZO condition was slightly greater than that of the ZI condition. It might be considered that the ZI condition placed a greater focus on an object more than the ZO condition. These results indicated that when the patients with USN concentrated on an object, their USN symptoms were more aggravated. The subjects'dressing, transferring, and locomotion of checklist by Halligan et al. indicated high percentage of presence of USN symptom [28]. Although the common BIT did not sufficiently show USN where the correct answer rate score of left space was more than 80%, the special test with HMD indicated USN where the correct answer rate score of the left space was about 60%. The HMD test may be able to better find a USN symptom which can not be easily discovered by the common clinical test.

In our former study, the use of the HMD improved the neglect symptoms in all subjects who had right cerebral hemisphere damage [32]. Rossetti et al. investigated the effect of prism adaptation on neglect symptoms, including the pathological shift of the subjective midline to the right [33]. They reported that all patients exposed to the optical shift of the visual field to the right were improved in their manual body-midline demonstration and on their classical neuropsychological tests. Lee et al. [34], Woo and Mandelmant [35] also suggested the effectiveness of the Fresnel prism when placed on a spectacle lens for improving various visual-field losses. The improvement induced by the HMD indicates that a signal is given to the brain that stimulates the natural recovery process in the same manner as the prism adaptation method. Moreover, the HMD system may lead to the further correction of left neglect than a Fresnel prism placed on a spectacle lens. Since a high power Fresnel prism membrane for obtaining a wide field of view is not clear, the prism produces a distortion of a real image and has lowered capabilities of visual acuity. By contrast, the HMD has the possibility of obtaining various fields of view without deterioration of visual acuity.

The HMD system has the advantage of being non-invasive, safe, and one can easily change the size of the visual field. Although the standard clinical examinations [36,37] were mainly used in a horizontal two-dimensional plane, the HMD system can easily produce a standard clinical examination related more closely to ADL in other planes, frontal or sagittal plane. On the other hand, the HMD system has to develop greater portability, a lighter weight and a decreased delay of response between the computer and the HMD regarding a transformation of data. The system's delay time is 50 m seconds. Therefore, the HMD system needs a higher level of technology of processing, recording and displaying a changed visual field of view in near or real time.

Technique of the HMD system may play an important role in the neuropsychological rehabilitation of unilateral spatial neglect as an evaluation device. Bowen et al. performed a systematic review of publish reports. They reported that 17 of which directly compared right brain damage (RBD) and left brain damage (LBD) and USN occurs more frequently after RBD than LBD was apparently supported by a systematic review of published data. However, an accurate estimate of the rates of occurrence and recovery after stroke could not be derived. They suggested that different USN disorders may exist, which may require type-specific rehabilitation approaches. Our system may have clinical implication for new assessment because HMD can change versatile visual input to fit each patient's degree of USN. Because, a clinical assessment for USN may be able to use various images in HMD by a computer such as change of colors and partial enlarge or reduce of real image, and to produce suitable visual information in HMD for each patient who has USN.

In this research, HMD evaluation could produce the condition of an object-centred allocentric co-ordinate. This means that our system can focus on the evaluation of the allocentric system to a greater degree than the egocentric system. A future study should be able to produce the condition of an egocentric system. In this case, a HMD display should be synchronized with a small CCD camera to be placed on the head or trunk. Moreover, eye and head movements should be measured in order for an analysis of eye – head or eye – hand coordination. It may be that eye and head movements are related to USN symptoms. In addition, we should identify the mechanisms behind the effectiveness of the HMD system and gather more from the patients.

In conclusion, the results showed that the assessment of USN using an HMD system may clarify the left neglect area which can not be easily observed in the clinical evaluation for USN. Moreover, it might be hypothesized that the USN test using HMD may display greater accuracy and be able to assess the occurrence and degree of USN more than the common clinical test. HMD can produce an artificially versatile environment ass compared to the common clinical evaluation.
